# What Types of Strategies Are Effective in Lumbar Spine Surgery? Considering the Etiology, Imaging Findings, and Risk of Complications

**DOI:** 10.3390/jcm12134443

**Published:** 2023-07-01

**Authors:** Shoji Seki, Koji Akeda, Takashi Kaito, Tetsuro Ohba

**Affiliations:** 1 Department of Orthopedic Surgery, Faculty of Medicine, University of Toyama, 2630 Sugitani, Toyama 930-0194, Japan; 2 Department of Orthopaedic Surgery, Mie University Graduate School of Medicine, Tsu City 514-8507, Japan; k_akeda@clin.medic.mie-u.ac.jp; 3 Department of Orthopedic Surgery, Osaka University Graduate School of Medicine, 2-2 Yamadaoka, Suita 565-0871, Japan; takashikaito@gmail.com; 4 Department of Orthopaedic Surgery, Faculty of Medicine, University of Yamanashi, 1110 Shimokato, Chuo 409-3898, Japan; tooba@yamanashi.ac.jp

Lumbar spine surgery is commonly performed worldwide for the treatment of lumbar spinal disorder, and the surgery saves many patients with lower back and lower extremity pain. Lumbar spinal disorder is common and leads to substantial morbidity and losses of productivity in society. Although there have been many reports on the pathophysiology of lumbar spinal disorder using the approaches of genetics, molecular biology, pathology and imaging, it remains unclear. It is thought that lumbar spinal stenosis (LSS), a lumbar spinal disorder, is due to lumbar ligamentum flavum (LF) hypertrophy. LF hypertrophy is partly caused by oxidative stress, and the effect of N-acetylcysteine (NAC) in suppressing it has been verified. It has been verified that p38, Erk, and p65 phosphorylation are involved in intracellular oxidative stress signaling in LF cells. NAC is likely a potential therapeutic agent against lumbar spinal canal stenosis [1]. Furthermore, LF hypertrophy not only causes LSS but may also cause the exacerbation of adolescent idiopathic scoliosis [2].

In recent decades, diagnostic imaging of the lumbar spine has made great strides, such as tractgraphy, CT/MRI fusion imaging, and whole-body bone X-rays. Advances in imaging technology have made blood vessels more visible, making it possible to read detailed anatomical findings from images to prevent complications. Gonadal vein (GV; testicular and ovarian vein) injury is a major vascular injury, and the incidence of ovarian vein injury was approximately 0.9% [3]. Despite the importance of blood vessels in preventing complications, their relationships to the iliopsoas muscles and vertebral bodies are still unclear. Therefore, it is necessary to pay particular attention to the anatomical position of the gonadal vein when performing lateral lumbar interbody fusion [4]. Proximal junction disease (PJD) and surgical site infection (SSI) are among the most common complications after spinal surgery. These risk factors are not fully understood. Therefore, imaging analysis using CT has shown that osteopenia and sarcopenia are associated with SSI [5]. Sarcopenia and osteopenia are independent risk factors for SSI and PJD after surgery. On the other hand, previous studies have shown that diffuse idiopathic skeletal hyperostosis (DISH) appears in 38% and 49% of cases in the thoracic and thoracolumbar regions, respectively, and is extended to the lumbar region in 13% of cases [6]. When choosing the extent of fixation and decompression for lumbar surgery, we must be very careful due to the risk of PJD if DISH is present. If lumbar decompression surgery is performed at the caudal end of DISH, decompression surgery without fusion, sparing the osteoligamentous structures at midline, should be considered the standard surgery for preventing PJD [7].

Conservative treatments for lumbar spinal disorders have thus far included pharmacological treatment, injections, rehabilitation, and surgical treatment using braces and are still being continued today. The proportion of the patients with LSS who underwent pharmacological treatment, such as the use of non-steroidal anti-inflammatory drugs, pregabalin/mirogabalin, opioids, prostaglandin E1 analogs, and neurotropin, significantly decreased after lumbar surgery [8]. People who do not show improvements in their walking abilities or social lives have higher risks of postoperative increases in the administration of the drugs mentioned above [8].

On the other hand, the surgical treatment of lumbar spinal disorders has progressed in recent decades. Microendocopic surgery of the lumbar spine is becoming more common worldwide. When multilevel decompression is required, microendoscopic bilateral decompression is associated with significant improvements in lower back and lower extremity pain compared with conventional microscopic decompression [9]. Multilevel decompression with the bilateral microendscopy in multiple spinal stenosis is likely to be more suitable than microscopic surgery. Regarding lumbar disc herniation, percutaneous transforaminal discectomy (PTED) had the obvious advantages of being less invasive and having a faster postoperative recovery, but the recurrence and revision rates were higher than those of microendscopic discectomy and traditional open surgery [10]. Additionally, for preventing complications of intraoperative hemorrhage, it has been used during spine surgery in adults and children [11,12]. However, severe fluctuations in blood pressure during spine surgery in the prone position may lead to fatal complications such as cardiac arrest, neuropathy, myocardial ischemia or infarction, acute kidney injury, and unilateral or complete vision loss [13,14]. Recently, an algorithm for the Hypotension Prediction Index (HPI) was developed by Edwards Lifesciences (Irvine, CA, USA). There is also a report that the use of the HPI algorithm significantly reduced the amount of bleeding compared to a group that did not use it [15].

In conclusion, as a strategy for lumbar spine surgery, the etiology and pathophysiology of the disease are first inferred [1,16], and the extent of the surgery is then determined based on spinal canal stenosis, the presence or absence of bony unions such as DISH [7], and the morphology of the fracture [17] based on image analysis [4,5,7]. Next, after selecting minimally invasive surgery [10], microendoscopic surgery [9,10], or conventional surgery as the surgical method, appropriate spinal instruments and cages [18] are selected. Finally, as a strategy for preventing complications, it is desirable to devise means of reducing the risk of bleeding due to hypotensive anesthesia [15], reduce psudoarthrosis [19], and reduce adjacent vertebral body fractures [20]. Appropriate prescriptions for relieving pain such as lower back and leg pain are also important for reducing postoperative complications [8]. Further research studies will progress in the future.
